# Temporomandibular chronic dislocation: The long-standing condition

**DOI:** 10.4317/medoral.21221

**Published:** 2016-10-01

**Authors:** Mariano Marqués-Mateo, Miguel Puche-Torres, Maria-Eugenia Iglesias-Gimilio

**Affiliations:** 1 MD, PhD, Associate Professor University of Valencia, Spain. Department of Oral and Maxillofacial Surgery, Hospital Clínico Universitario, Valencia, Spain; 2 MD, PhD, Associate Professor University of Valencia, Spain. Head of Department of Oral and Maxillofacial Surgery, Hospital Clínico Universitario, Valencia, Spain; 3 Department of Oral and Maxillofacial Surgery, Hospital Clínico Universitario, Valencia, Spain

## Abstract

**Background:**

The temporomandibular joint (TMJ) dislocation can be categorised into three groups: acute, habitual or recurrent and long-standing. The long-standing or protracted lower jaw dislocation refers to a condition that persists for more than one month without reduction. There are a great variety of methods for its treatment, from the manual or non-surgical, to surgical ones like the indirect approach (conservative surgical approach) and direct approach (open joint). Additional procedures in unsuccessful cases may include extra-articular orthognathic techniques to correct a malocclusion until joint replacement.

**Material and Methods:**

We report four new cases with a minimum of 6 weeks dislocation who were seen since 1995 to 2015 in the Maxillofacial Department of the Clínico Hospital (Valencia, Spain), in which the mean age was 57.5 years. Most of them were bilateral and the gender was predominantly female. Additionally, we have reviewed the related literature.

**Results:**

All of the cases were successfully treated and half of them required open surgery.

**Conclusions:**

The report confirms the difficulty of the treatment and reaffirms the necessity to bear in mind the wide variety of methods available for the treatment of this pathology. We stress the difficulties associated with managing the treatment and of suggesting new guidelines. The best option still remains not to delay the diagnostic and to select the appropriate initial treatment.

**Key words:**Temporomandibular luxation, TMJ dislocation, protracted dislocation, long-standing dislocation.

## Introduction

A luxation (or dislocation) is a condition in which a joint is displaced from its articulations, such as by an excessive movement of the condyle beyond the articular eminence, with complete separation of the articular surfaces and fixation in that position, i.e., open locked. The patient alone cannot return it to its initial position as it requires external manipulation (1,2).

The temporomandibular joint (TMJ) dislocations may be further subdivided into acute or chronic conditions: chronic recurrent if it appears repeatedly over a short period of time; chronic persistent dislocations if it persists over a long period of time (1,2). Chronic persistent dislocation can be defined (3) as acute dislocation left untreated or inadequately treated for 72 hours or more, and there is consensus that if the situation persists for more than a month, it is labelled a long-standing or protracted temporomandibular joint dislocation. This last condition is the most challenging and difficult to treat of the three. Various terms have been proposed for this condition: persistent (3), protracted (4), prolonged (5), or long-standing (3), and they may be used indifferently. This is totally different from recurrent chronic or habitual dislocation, where the patient suffers many frequent episodes of acute luxation that are treated satisfactorily, although it could involve some surgical procedures.

Dislocation of the TMJ occurs in up to 7% of people during their lifetime. TMJ dislocation represents 3% of all articular body luxation (6). The etiology is predominantly trauma in 60% of cases 2). The problem can affect any age but is most common in 3rd and 4th decades (5). In general, TMJ lux•ation is found in females (7), but some authors report a higher incidence in men (5).

The pathogenesis of TMJ dislocation is multifactorial. It occurs frequently as a result of the following situations or procedures (2,7):

- Altered structural components: lax capsule, weak ligaments, steep eminence, abnormal condylar shape, and atypical disc position.

- Predisposing factors (partial list):

• Systemic disease (Ehlers-Danlos/connective tissue disease, neurodegenerative /neurodysfunctional diseases [i.e., Huntington disease, epilepsy, Parkinson disease, multiple sclerosis], and muscle dystrophies/dystonias).

• Pharmacologic: Use of phenothiazines or metoclopramide (extrapyramidal effects of their use).

- Other associated factors:

• Iatrogenic: intubation/laryngoscopy, dental, ear, nose and throat procedures, gastrointestinal endoscopies.

• Spontaneous: laughing, yawning, vomiting, or singing.

• Trauma.

These afore-mentioned factors mainly determine the type and direction of the dislocation. In addition, age, dentition, cause and duration of the dislocation as well as the function of the masticatory muscles contribute significantly to the mechanism and management of temporomandibular joint dislocation (2,8).

The capsule of the joint is the most important structure which stabilizes the joint reinforced by the lateral ligaments (2). This dense capsulo-ligamentous apparatus functionally restricts extremes of mandibular movement. It is generally accepted that initial dislocation is caused by lack of muscular coordination during the initiation of jaw closure (9), and it occurs as a combination of 3 factors: laxity of ligaments, the size of the bony eminence and muscular spasm.

Although the most common condylar dislocation is anterior to the articular eminence, onto the preglenoid plane, the condyle can be dislocated in a medial, lateral, posterior, or intracranial position (9).

Clinically the condition is characterised by the inability to close the mouth after wide opening, and change in occlusion with open bite and/or lateral mandibular deviation. Palpation in the preauricular region reveals an empty joint fossa and may reveal the condyle anterior to the joint.

The pain and discomfort associated with this condition leads the patient to seek immediate treatment. If the patient does not, it becomes a chronic luxation.

Of the three different conditions, a dislocated jaw that prolongs or sustains the luxation is the rarest. Out of 128 articles reviewed by Akinbami (2), there were 79 cases of acute luxation, 311 cases were chronic recurrent TMJ dislocations, and only 35 cases were chronic long-standing ones. There are few series to show what this rare condition represents in the total of TMJ luxations, ranging from 8.2% (2) to 30.2% (5).

If this condition persists beyond 6 months, it is labelled an extra long-standing luxation. In these cases, a pseudo-joint is formed so the patient can have an adequate functional movement. For this reason, only minimal attempts at treatment were made or there was no treatment given at all. It is a very rare condition, Caminiti and Weinberg (1) have reported only four of them over the last twenty years.

## Material and Methods

The present paper reports four cases of chronic anteromedial TMJ dislocations seen over a period of 20 years (1995 to 2015) in our Department. In every case, the mandibular dislocation persisted for a minimum of 6 weeks, thus being considered protracted or long-standing dislocations.

This manuscript has been reviewed and approved by the Institutional Review Board of the Clínico Hospital of Valencia (Spain).

Case 1.

70 year old female patient who was referred to our clinic in 1995 presented with a dislocated jaw maintained during 6 weeks of evolution, without a previous history of trauma or dislocation. She was edentulous. Orthopantomography revealed a previous bilateral dislocation. Closed reduction under the effect of local anesthetic around the joint and muscle relaxants were performed without success. Consequently, she underwent surgery, and the reduction of the bilateral dislocation was done under general anesthesia by pulling down on wires from both mandibular angles. After 5 years she had not presented with another episode.

Case 2.

A 34-year-old female came to our clinic in 2005 presenting with myofascial pain at the level of the bilateral mandibular ramus and with a history of intervention in another hospital for mandibular lateral deviation six years before. No history of trauma, or acute pain was given. The patient‘s occlusion was worsening, with poor intercuspation in centric relation. The opening was slightly decreased. In the radiographic study by ortopantomography revealed a right mandibular condyle out of the glenoid fossa and anterior to the temporal eminence. A bilateral low condylotomy had probably been performed previously.

The patient did not improve after occlusal rehabilitation. Consequently, she underwent surgery utilising a double approach: retromandibular via Risdom and preauricular; and a condylotomy with an attempt to reposition the condyle in its fossa, just managing to place it at the level of the temporal eminence. The condyle was fixed with miniplates. The pain subsided after a few months, and the patient is currently asymptomatic with normal occlusion. A follow-up of 8 years has shown no alterations in occlusion.

Case 3.

A 76-year-old female, totally edentulous, presented in 2012 with a bilateral anterior dislocation with eight weeks of evolution. The patient awoke one morning with pain in both TMJ and with an occlusal denture mismatch. She was living in a small town, and once well-diagnosed, was sent to a specialist.

Manual reduction was attempted with muscle relaxants, but was unsuccessful. Subsequently, in the operating room and under general anesthesia, dislocation reduction was achieved and the dental prosthesis were screwed into the jaws to keep the normocclusion and mandibular condyles within their respective cavities. The fixation remained wired for three weeks. At 36 months, the patient had had no new dislocation episodes.

Case 4.

**Figure 1:**
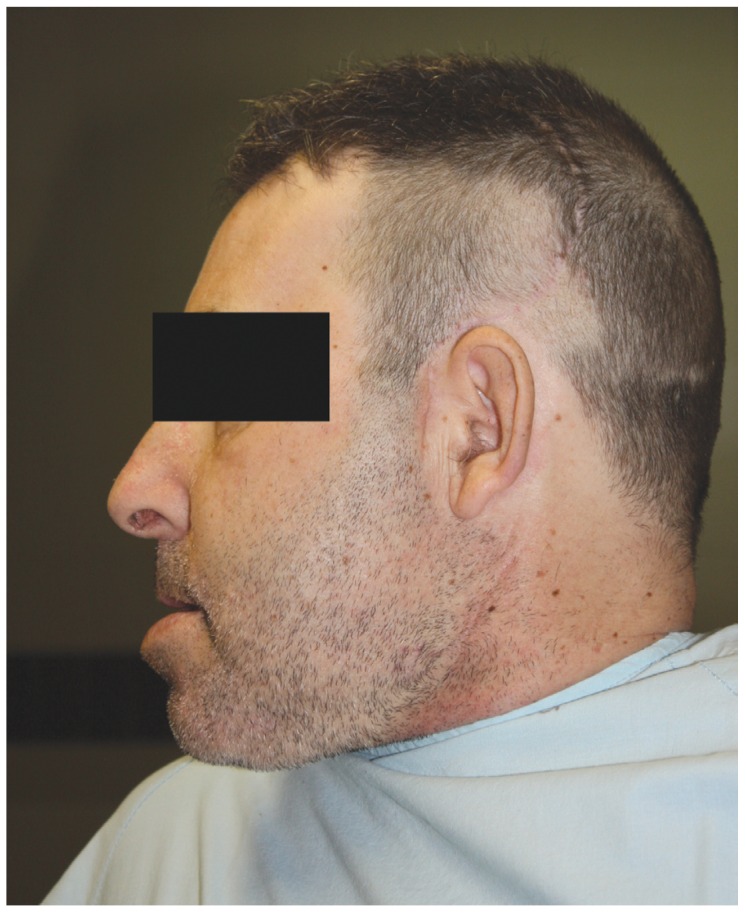
Case 4. Before treatment to correct the long-standing dislocation.

**Figure 2:**
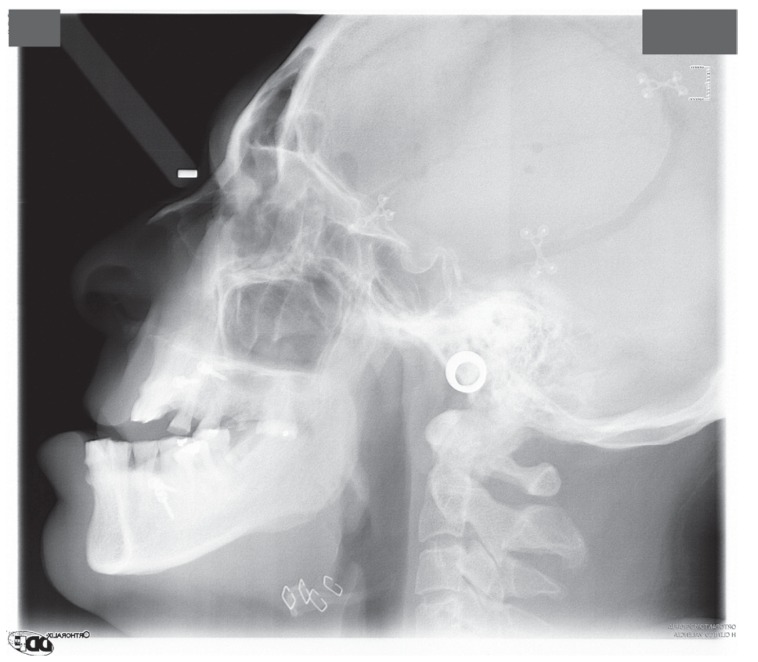
Telerradiography before open treatment. The old neurosurgery approach and the angle approach can be observed.

A 50-year-old male, referred to our clinic in 2014, presented with alterations in the occlusion after neurosurgery. Four months previously he had undergone a surgical intervention for a left brain tumor of the temporal lobe. Immediately after surgery, he exhibited an alteration to his form of chewing that was not taken into account by his physicians. He had an accommodative occlusion, and after four months it reappeared. Consequently, he was referred to our service. In a partially edentulous patient, radiography revealed occlusal wear facets of upper and lower incisors and mandibular bilateral anterior dislocation. He had a brachycephalic pattern and a class III prognathic profile. (Figs. 1,2)

**Figure 3:**
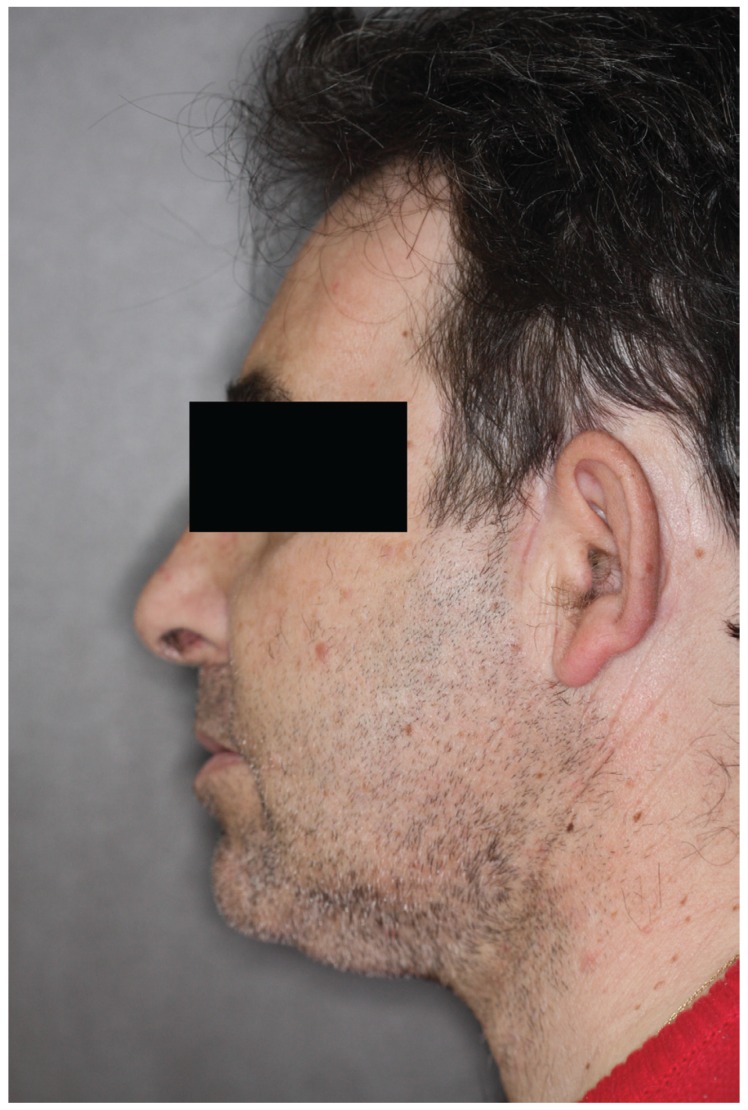
Case 4. After open treatment: bilateral eminectomy, removal of fibrosis and high condylectomy.

A closed reduction was attempted after the administration of local anesthetics and muscle relaxants to the temporomandibular joint, with unsatisfactory results. A closed reduction was attempted again, this time under general anesthesia, followed by open reduction with indirect traction wires in both mandibular angles and the placement of intermaxillary fixation with elastics. Radiography revealed that the condyles were not correctly placed. Consequently, open surgery of the joint via a bilateral pre-auricular approach was performed. An edge-to-edge occlusion was finally achieved after meniscectomy, eminectomy, a high condylectomy, and with the removal of fibrous tissue and reducing the dislocation. Intermaxillary fixation was placed for 2 weeks (Fig. 3). The patient has been asymptomatic for one year.

## Discusion

Temporomandibular joint dislocation is a common presentation that is usually reported as an acute episode of unilateral or bilateral displacement of condyles anterior to articular eminence. In complete dislocation, condyles lie completely out of the glenoid fossa. Often, failure to diagnose or inappropriate treatment in the initial stage results in prolonged malposition of an acutely displaced condyle leading to chronic protracted dislocation (10). Untreated cases of acute TMJ dislocation become chronic.

An acute TMJ dislocation of more than four weeks can be called a long-standing, protracted or persistent dislocation. It is a rare condition. Gottlieb (11) presented 24 cases in 1952. In 1986, Wijmenga *et al.* (4) presented three new cases of long-standing dislocation of the TMJ and reviewed 37 cases. Since then, only one long serie, by Ugboko (5) (29 cases) a meta-analysis, by Akinbami (2) (35 cases), and a few isolated case reports have been presented.

In the present study, all the cases had a dislocation for more than four weeks. The average age was 57.5 years, similar to the 52.7 reported by Caminiti and Weinberg (1) and the 62.8 years by Huang (3), ( Table 1). Rattan *et al.* (12) reported a mean age of 47.7 years. The pathology in our serie is more frequent in women, in agreement with what some authors have reported (1,7), while contrary to that for other ones (3,5). It is clear that there is no consensus about gender. Nevertheless, this pathology appears more frequently in older people than does acute dislocation.

**Table 1:**
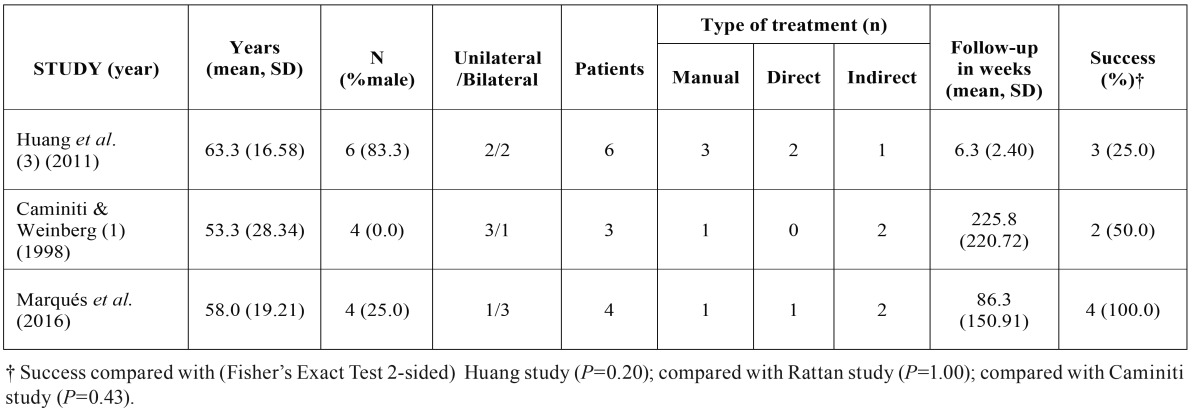
Comparisons of reported series.

Published studies (5) show that in the majority of cases, this clinical subtype is due to yawning in women. In men, however, the causes are varied, including staying in an intensive care unit (3). Trauma was not a decisive factor for this subtype.

Bilateral anterior dislocations were the most frequent in all series (2,3,5). In ours, the long-standing dislocation etiologies were muscular incoordination in two cases, anesthetic manipulation in another, and sequelae of an orthognathic surgery in the last. There was no association with trauma. Three were bilateral. Out of 425 dislocations, Akinbami (2) report only four cases of unilateral dislocation.

A history of dislocation of more than one and a half months was a result of the delay in treatment in cases 1 and 3, inadequate treatment in case 2, and of the failure to diagnose it in case 4. No history of trauma was presented, only a previous extra-articular surgery. All our cases were treated successfully. That notwithstanding, in other series (3,5) there were patients for whom minimal procedures did not reduced the luxation, and they did not accepted a more aggressive technique. Alternately, the time which had passed was too long for the use of a minor technique, and the patient refused surgery.

According to Rattan *et al.* (12), manual manipulation and indirect traction techniques were usually unsuccessful, but patients needed be treated by these methods before the use of other more aggressive ones. Shakya (7) only reported one case of long-standing dislocation, and he achieved a manual reduction although 4 months had passed. That has not been the usual outcome. We agree with the authors that even though in long-standing cases the probability that the use of aggressive methods is greater, it is necessary to utilize conservative techniques first. More aggressive one should be reserved only for when one is convinced they are necessary (2,7). It is difficult to evaluate the results in others series because the number of procedures in each joint and the relationship joints/number of patients is not well defined. There have been various techniques used in the same patient that have not been well reflected in the documentation.

Anterior dislocations are the most common (four of four in our report) and occur as a result of the displacement of the condyle anterior to the articular eminence of the temporal bone. They are usually secondary to an interruption in the normal sequence of muscle action when the mouth closes from extreme opening. The masseter and temporalis muscles elevate the mandible before the lateral pterygoid muscle relaxes resulting in the mandibular condyle being pulled out of the glenoid fossa and anterior to the bony eminence (13).

Ideally, acute mandibular dislocation requires immediate effective reduction, which can usually be accomplished with a manual closed technique described centuries ago by Hippocrates.

After 2 weeks, spasms and shortening of the temporalis and masseter muscles occur and reduction becomes difficult to achieve manually. In spite of this, it can be first treated by manual manipulation (closed reduction). The reflex spasm of the muscles prevents reduction manoeuvers; in such cases, local anesthesia can be used. It is based on the theory that the dislocation is maintained by muscle spasm, a result of the painful stimuli arising from the capsule. Local anesthesia surrounding the joint, serving as auriculotemporal nerve blocks, can be used. It may be assisted by sedation using intravenous diazepam or the use of general anesthetics (7).

The duration of the dislocation plays an important role for prognosis. Delays in treatment can lead to spasm of the masticatory muscles as well as fibrosis and scarring of the retro- and peridiscal tissue due to intra- and extra-articular haemorrhage in post-traumatic cases (12).

For chronic and recurrent dislocations, different management options have been divided into non-surgical (conservative approach), and surgical methods (indirect approach and direct approach). Gottlieb (11) in 1952 reported only three of 24 long-standing cases that were successfully reduced by manual reduction. Only one in our series was. Adekeye *et al.* (14) manually reduced four out of 24 cases.

Despite these attempts, most cases are responsive to surgical procedures (open reduction) (2). This leads to the commencement of chronic protracted dislocation. Due to the rarity of this condition, surgeons may have limited experience in its management, and no standard guides of evaluating or treating it were documented until Rattan *et al.* (12) and Caminiti (1) did so.

The surgical goals must continue to be the following (12):

1- Reduction,

2- Functionality - Normocclusion,

3- Minimal morbidity and sequelae (risk of ankylosis), and,

4- No recurrence.

The next level is the indirect open procedure, and it can be done with a bone hook used to apply traction via the sigmoid notch or by traction with wires through holes drilled in the angle of the mandible (15,16).

It may be necessary to persevere in the manipulation because the condyle may need some attempts. The arch bar or IMF (intermaxillary fixation) with screws for elastic traction should be applied to prevent any recurrence following reduction. IMF screws can be used in edentulous patients or partially edentulous patients. In addition to their preventive function, they can be used to fix dentures, as in our case 3. Moreover, IMF and anterior elastics require posterior bite blocks (12).

The manual or conservative surgical approach was sufficient in from 33% (3), to 54% (2), similar to our series: 2 of 4.

Any attempt to reduce the luxation with closed manipulation in patients with more than 4 weeks was unsuccessful by Huang (3). These cases should be solved by open reduction, either indirect or direct. When reducing the dislocated condyles via the indirect open procedures, and it fails, direct reduction of dislocated condyles via a preauricular approach is the following level of procedure. Direct or invasive interventions are aimed at anatomic modification of the eminence, condyle or musculocapsular tissues (9). At this point, different authors have chosen different solutions to the problem.

When surgery is indicated for chronic protracted dislocation, especially in cases of longer duration, the main goal may be to reposition the condyle in the glenoid fossa and restore movement.

Open surgery in the joint was necessary in from 16% (3), to 54% (5). Fifty per cent of our cases required open joint surgery. One of them was the case of protracted luxation for more than six years, while the other was a brachycephalic pattern with a great muscularity. We want to point out that the two cases which required open surgery were the longest ones. Even though all our cases were treated successfully, in this subtype of dislocation there is a respectable number of patients, between 17% (5) and 50% (3), who are not treated or refuse the treatment when open techniques are proposed, see  Table 1. The success of treatment is not significant with compared with others due to lower case letters. There are different techniques for the direct approach to the articulation. Most of them use an open approach, usually via the pre-auricular region (7). Myrhaug first reported the eminectomy in 1951 (17) as a treatment for dislocation. Removal of the eminence will facilitate the return of the condyle without any intrusion into the glenoid fossa: This is one of the most valuables techniques known.

Various other methods, such as condylotomy and myotomy have been tried (7). Myotomy with resection of the insertion of the external pterygoid muscle was described by Bowman in 1949. The inconvenience of this procedure is that it limits the mandibular translation and allows only rotational movement of the condyle. Meniscoplasties and meniscectomies are relevant procedures done when altered disc morphology and position cause dislocation or prevent self-reduction (2). Nevertheless, direct joint exposure and manipulation can predispose to ankyloses.

Managing the malocclusion without operating in the TMJ is the second goal when the first is not possible. This can be accomplished utilizing such conventional intraoral osteotomies as a sagittal split osteotomy or a vertical ramus osteotomy (9). These can be used to achieve an adequate functional occlusion and improved aesthetics. Adekeye *et al.* (14) described a technique in which the occlusion was restored by means of an inverted L-shaped ramus osteotomy. Other types of osteotomy should not be considered (for example, vertical and horizontal ones) because of the possibility of interference with the coronoid process. The bilateral sagittal split technique offers a better outcome for the correction of the occlusion because there is no extraoral scar. Akinbami (2) reported orthognathic procedures in 34% of these patients.

Lee *et al.* (18) and Rattan *et al.* (12) have proposed the midline mandibulotomy in difficult TMJ dislocation, but it requires integrity of the joint, whit no fibers inside that could do to fail the technique. Etiology no traumatic is the indication. In the new algorithm proposed by them this technique is used before open procedures.

Most agree that the longer the dislocation, the more difficult the reduction of the condyle becomes. Despite having limited experience in treating long-standing dislocation of more than 3 months they suggested that when long-standing dislocation has persisted for 4-12 weeks it is best treated by open reduction. Their findings suggest that surgical procedures are probably necessary to correct dislocations greater than 3 months (3). Our results agree with the literature we had to use open surgery in the joints for luxations of more than 8 weeks. To that we add that the more aggressive technique had to be used.

Total joint replacements should be considered when all appropriate treatments fail in chronic protracted cases, especially in those with associated degenerative joint diseases, in order to recover proper TMJ function and anatomy. It constitutes the highest level of treatment.

In the case the dislocation persists for an extended period of time, a pseudo-joint is formed, and the patient can have adequate functional movement. Since some patients reject further interventions, the articular fossa gets filled with fibrous tissue which eventually hinders the condyle seating in its normal position. Caminiti and Weinberg (1) report four cases of extra-long standing dislocation in which they demonstrate the difficulty of treatment. In cases of chronic dislocation, where the joint has been out of the fossa for more than 6 months, it is important to consider orthognathics or total joint prosthesis (9). Adekeye *et al.* (14) emphasizes the difficulties encountered in adequately reducing the condyle. The second case presented matches that opinion.

In long-standing TMJ dislocations, almost all recommendations indicate that the surgeon should treat the patient using a step-by-step approach, starting with a minimally invasive conservative technique and progressing to more invasive surgical procedures (12).

Akinbami (2) has proposed a new classification based on the condyle head position: below (I), in front (II), and in front high up (III). Moreover, there is an algorithm of procedures based on it. For the third type (protracted dislocation), though, it does not offer a unique technique.

A protocol for the management of long-standing TMJ dislocations has been advocated (12). It starts with non-surgical procedures, followed by indirect open access and then on to direct articular access. There is not, however, any explanation of the different methods of open surgery nor their preferences. Having described the various treatment options, it is up to each diagnostician to decide at each level ( Table 2) which is the first option and which the next if necessary. The literature review elucidates the troubles surgeons face, the need for a sequence or combination of different techniques and the unpredictability of the treatment. It offers an idea of the difficulties associated with standardising guidelines or protocols.

**Table 2:**
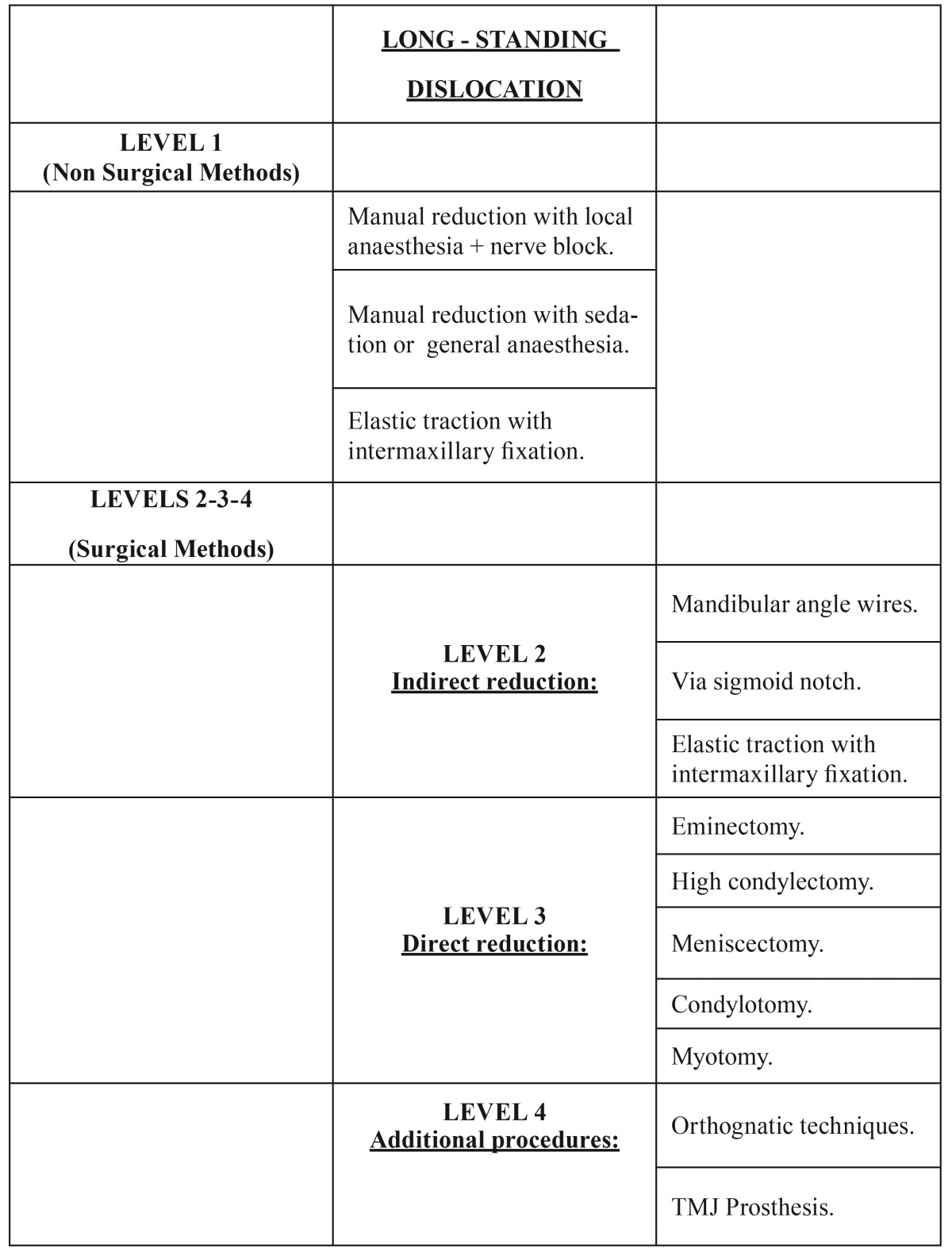
Surgical treatment procedures for luxation of TMJ. Modified from Shakya.

According to Akinbami (2), the main factor for correct joint replacement is the elapsed time to treatment. As this increases, the prognosis worsens. Consequently, after 3 months the success rate drops dramatically. The second factor to consider when planning treatment is etiology. That notwithstanding, Rattan *et al.* (12) opined that the etiology of the dislocation (trauma or non-trauma) is the primary prognostic factor, and duration is secondary. We do not consider trauma to be a main factor because it is so infrequent.

## Conclusions

The best way to handle cases of TMJ disorders is to treat them as early as possible to avoid further complications. This increases the possibilities of success. It is necessary to keep in mind that if the dislocation persists longer than 4 weeks, the long-standing or protracted condition, treatment will be more difficult and possibly unsuccessful.

The first objective is to restore joint anatomy and if it is not possible, the second one is to achieve a functional occlusion.

Reviewing the treatments of the cases submitted, there are levels of treatment, but no definitive techniques. It is advisable to start with non-surgical techniques, followed by other minimally invasive ones (normally indirect) and then by performing open joint surgery. If, despite the above, success is not achieved, can be attempted with the ultimate aim of achieving a proper mandibular occlusion.
